# Purification and characterisation of natural Cor a 14, the 2S albumin from hazelnut, and its isoforms

**DOI:** 10.1186/2045-7022-5-S3-P18

**Published:** 2015-03-30

**Authors:** Sabine Pfeifer, Pawel Dubiela, Merima Bublin, Christine Hafner, Karin Hoffmann-Sommergruber

**Affiliations:** 1 Department of Pathophysiology and Allergy Research, Medical University of Vienna, Vienna, Austria; 2 Karl Landsteiner Institute for Dermatological Research, St. Poelten, Austria

## Background and aim

Allergens from nuts are known to often induce severe allergic reactions in sensitive individuals. Nuts are a nutrient dense food and a good source of unsaturated fatty acids and other lipids. The allergenic properties of hazelnut allergens could be altered by binding to or being in close proximity to these lipids. The aim of this study was to purify and characterize natural Cor a 14, the 2S albumin from hazelnut, and to investigate whether and how hazelnut lipids affect its allergenic activity.

## Methods

Cor a 14 was purified from raw hazelnuts using a combination of precipitation and chromatographic techniques. The protein was identified using proteomic approaches such as 2D-gel electrophoresis, 1D-NMR, mass spectrometry, and de novo sequencing. Lipids were extracted using hexane and chloroform as a solvent. Protein-lipid fractions were prepared by mixing the purified allergens with lipids using a mass ratio of 1:2. The allergenic activity of purified Cor a 14 with and without hazelnut derived lipids was studied in *in vitro* IgE binding assays (ELISA, immunoblot), followed by RBL assays using sera from hazelnut allergic patients. Also digestion assays, simulating gastric and duodenal fluid, were performed.

## Results

In SDS-PAGE, purified Cor a 14 isoforms provide single bands of about 14 kDa. After reduction they split into two smaller bands, typical fo the two chains of 2S albumins. Using MALDI-TOF MS peptide analysis we could identify both subunits with a sequence-coverage of 100% and a molecular mass of ~12 kDa which is in accordance with mature Cor a 14 (UniProt: D0PWG2). 2D-electrophoresis suggests the evidence of additional isoforms. Digestion experiments revealed that native Cor a 14 is of intermediate stability. Native as well as denatured protein was recognized by IgE from sera from hazelnut allergic individuals. A dose-dependent release of β-hexosaminidase from RBL cells did not change when lipids were added to purified Cor a 14.

## Conclusions

We identified different isoforms of Cor a 14 from hazelnut. In contrast to other 2S albumins Cor a 14 displays intermediate stability under gastric and duodenal conditions. Addition of hazelnut lipids had no significant effect on the allergenicity of Cor a 14.

